# Does rhythm matter in acute heart failure? An insight from the British Society for Heart Failure National Audit

**DOI:** 10.1007/s00392-019-01463-5

**Published:** 2019-04-08

**Authors:** Simon G. Anderson, Ahmad Shoaib, Phyo Kyaw Myint, John G. Cleland, Suzanna M. Hardman, Theresa A. McDonagh, Henry Dargie, Bernard Keavney, Clifford J. Garratt, Mamas A. Mamas

**Affiliations:** 1 Division of Cardiovascular Sciences, Faculty of Biology, Medicine and Health, The University of Manchester, Manchester, UK; 2 Department of Cardiology, North West Heart Centre, University Hospitals of South, Manchester, UK; 3 The George Alleyne Chronic Disease Research Centre, Caribbean Institute for Health Research (CAIHR), The University of the West Indies, Bridgetown, Barbados; 4 Keele Cardiovascular Research Group, Centre for Prognosis Research, Institute for Primary Care and Health Sciences, University of Keele and Royal Stoke Hospital, Stoke-on-Trent, UK; 5 Institute of Applied Health Sciences, University of Aberdeen, Aberdeen, UK; 6 Robertson Centre for Biostatistics and Clinical Trials, University of Glasgow, Glasgow, Scotland, UK; 7 National Heart and Lung Institute, Imperial College, London, UK; 8 Clinical and Academic Department of Cardiovascular Medicine, Whittington Hospital, London, UK; 9 Faculty of Life Sciences and Medicine, King’s College London, London, UK; 10 Institute of Cardiovascular and Medical Sciences, University of Glasgow, Glasgow, UK

**Keywords:** Acute heart failure, Atrial fibrillation, Prognosis, Mortality

## Abstract

**Background:**

Atrial fibrillation (AF) is the most common sustained arrhythmia in patients with acute heart failure (AHF). The presence of AF is associated with adverse prognosis in patients with chronic heart failure (CHF) but little is known about its impact in AHF.

**Methods:**

Data were collected between April 2007 and March 2013 across 185 (> 95%) hospitals in England and Wales from patients with a primary death or a discharge diagnosis of AHF. We investigated the association between the presence of AF and all-cause mortality during the index hospital admission, at 30 days and 1 year post-discharge.

**Results:**

Of 96,593 patients admitted with AHF, 44,642 (46%) were in sinus rhythm (SR) and 51,951 (54%) in AF. Patients with AF were older (mean age 79.8 (79.7–80) versus 74.7 (74.5–74.7) years; *p* < 0.001), than those in SR. In a multivariable analysis, AF was independently associated with mortality at all time points, in hospital (HR 1.15, 95% CI 1.09–1.21, *p* < 0.0001), 30 days (HR 1.13, 95% CI 1.08–1.19, *p* < 0.0001), and 1 year (HR 1.09, 95% CI 1.05–1.12, *p* < 0.0001). In subgroup analyses, AF was independently associated with worse 30-day outcome irrespective of sex, ventricular phenotype and in all age groups except in those aged between 55 and 74 years.

**Conclusion:**

AF is independently associated with adverse prognosis in AHF during admission and up to 1 year post-discharge. As the clinical burden of concomitant AF and AHF increases, further refinement in the detection, treatment and prevention of AF-related complications may have a role in improving patient outcomes.

**Electronic supplementary material:**

The online version of this article (10.1007/s00392-019-01463-5) contains supplementary material, which is available to authorized users.

## Introduction

Atrial fibrillation (AF) is the commonest sustained arrhythmia in patients with heart failure (HF) with a prevalence reported between 30–50% in contemporary studies [1–4]. Previous studies have shown that the presence of AF in patients with HF is associated with an adverse prognosis, although many of these studies have reported this association in patients with chronic stable HF [5]. A meta-analysis including 50,000 patients suggested that the presence of AF was associated with increased mortality risk in both randomised controlled studies and observational studies, irrespective of left ventricular (LV) function [6]. It appears that the risk is greatest in patients with incident AF compared to those with prevalent AF, RR: 2.21 versus 1.19, respectively [7]. However, the prognostic impact of AF in patients admitted with an acute heart failure (AHF) is less clear [8–10].

Analysis of 10,701 patients hospitalised with an AHF as part of the EuroHeart Failure survey suggested that the presence of chronic AF did not impact in-hospital mortality (OR 0.84; 95% CI 0.69–1.00) although new-onset AF was an independent predictor of in-hospital mortality (OR 1.53, 95% CI 1.14–2.06) [11]. In contrast, in an analysis of 99,810 patients from 255 sites admitted with HF enrolled in Get With The Guidelines-Heart Failure (GWTG-HF) program in the United States, the presence of AF was an independent predictor of in-hospital mortality (OR 1.17, 95% CI 1.05–1.29; *p* < 0.005) [7], whilst one other study has shown AF to be associated with worse outcomes in patients with AHF with underlying ischaemic heart disease (IHD) only [12]. Previous studies have not reported whether the prognostic impact of AF in patients admitted with an AHF is similar in patients with HFREF (Heart Failure with Reduced Ejection Fraction) and HFPEF (Heart Failure with Preserved Ejection Fraction), across genders or different age groups or have only reported in-hospital mortality outcomes with no post-discharge outcomes studied [7, 13–15].

We have, therefore, studied the association between AF and in-hospital and longer term mortality outcomes in an unselected cohort of patients admitted with an AHF in England and Wales through analysis of the National Heart Failure Audit. Furthermore, we aimed to examine whether the observed association differs in patients with HFPEF and HFREF.

## Methods

The current study dataset is derived from around 150,000 patients hospitalised with heart failure and thus provides an excellent opportunity to study the clinical characteristics and outcomes in a ‘real-world’ setting. Mortality tracking is undertaken by the Medical Research Information Service using a patient’s National Health Service (NHS) number, which provides a unique identifier for any person registered with the NHS in England and Wales, and the Office for National Statistics.

### Study population

The National HF audit (NHFA) established in 2007 to monitor and improve care and treatment of patients is one of the largest HF cohorts in the world. 145 out of 150 NHS Trusts in England and Health Boards in Wales (97%) submitted data to the audit between April 2012 and March 2013. The audit collects information from unscheduled individuals who have been admitted to participating hospitals with a primary death or discharge with a coded diagnosis of heart failure. This is designated by the following ICD codes: I11.0 hypertensive heart disease with (congestive) heart failure; I25.5 ischaemic cardiomyopathy; I42.0 dilated cardiomyopathy; I42.9 cardiomyopathy, unspecified; I50.0 congestive heart failure; I50.1 left ventricular failure or I50.9 heart failure, unspecified. The National Heart Failure Audit has developed a minimum data standard, in an attempt to ensure that the records submitted to the audit are fit for purpose.

For patients with more than one reported hospital admission, we randomly selected one admission. The analyses were restricted to patients with a diagnosis of atrial fibrillation or sinus rhythm on an ECG performed during the admission. Patients aged less than 18 years, duplicate records, no ECG data to clarify rhythm status and missing outcome data were excluded from the study (supplement Fig. 1). Evident moderate or severe left ventricular systolic dysfunction are derived from results of echocardiography, or other gold standard tests (including MRI, nuclear scan) during the current admission or in the 12 months prior to admission. Death was defined as mortality from any cause. Patients aged younger than 18, with missing records of ECG data or outcome data and those with no life status recorded were excluded.

**Fig. 1:**
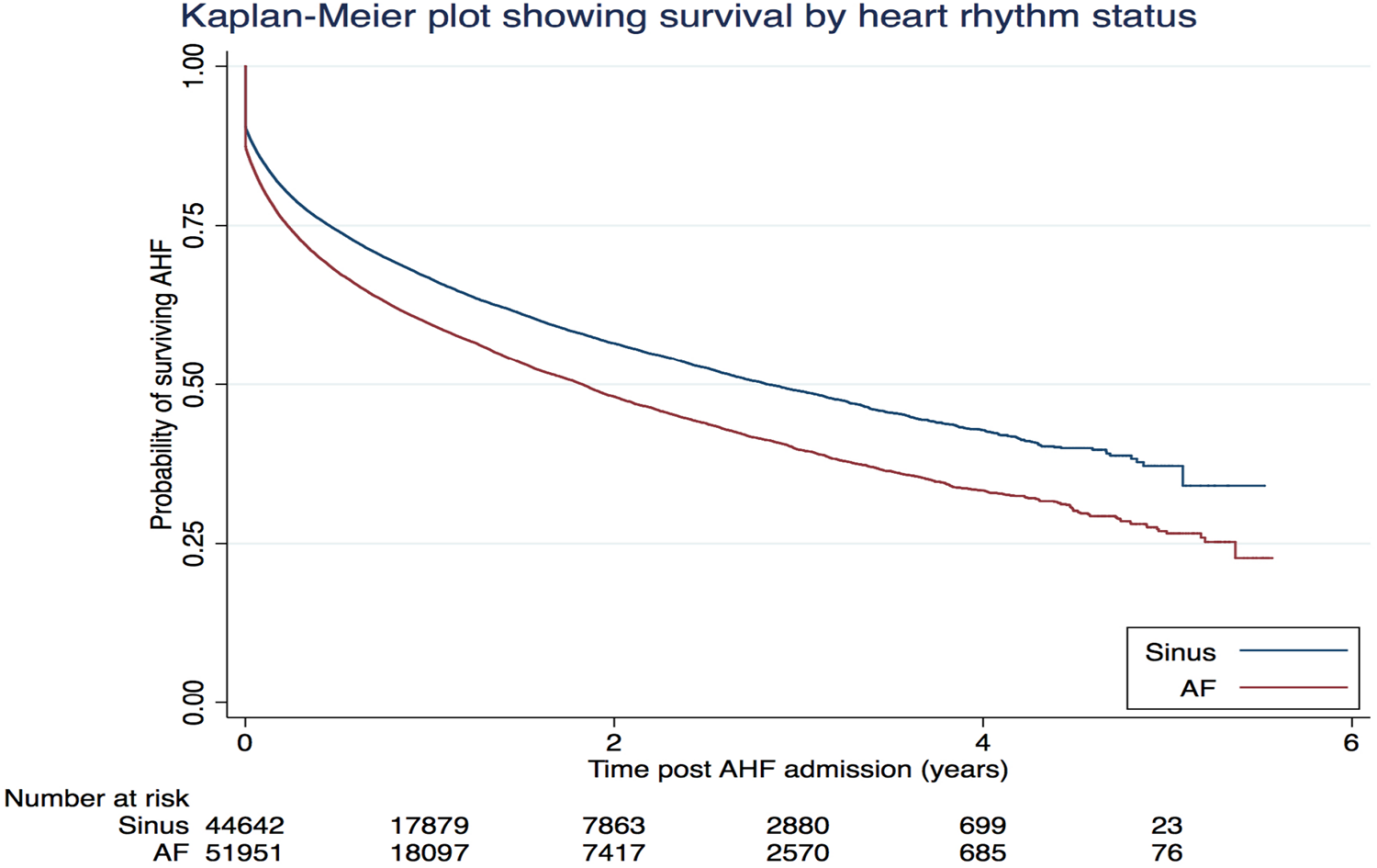
Crude KM survival estimates

### Primary outcome

The primary outcomes were all-cause mortality as an inpatient, at 30 days, 1 year and all deaths.

### Covariates

The National Health Service (NHS) information centre designed a secure, encrypted and web-based database called Lotus Notes for the recording of data relating to patients with heart failure which consisted of 233 fields. For the purpose of NHFA, 21 fields were assigned as core fields from this database and seven fields contained basic demographic information such as patient unique identifiers, gender and date of enrolment. The data were collected and recorded with the help of heart failure specialist nurses during each admission from patient case notes. Full details about selection of variables in this dataset and NHFA list of core fields are published elsewhere [16, 17].

### Statistical methods

The analysis was performed using the Stata/MP 13.1 statistical software (College Station, TX). The cohort was stratified into two groups, those with sinus rhythm and those with atrial fibrillation. Where an individual had multiple admissions we randomly selected a single hospital episode. Continuous variables are presented as mean or 95% confidence intervals (CI), or median (interquartile range). Categorical variables are presented as counts (%). We tested for differences between the groups using Chi-squared tests for nonparametric data and Student’s *t* test for normally distributed continuous variables.

We estimated hazard ratios (HRs) for mortality within 30-day using Cox regression with shared frailty or cluster models. A shared-frailty model is the survival-data analog to regression models with random effects and is used to model within-group correlation. Each hospital was assigned as a group variable within the model.

### Covariates in multivariable models

Multivariable adjustment included the continuous variables age as well as index of multiple deprivations and dichotomised categorical variables (yes versus no): sex (female versus male), history of myocardial infarction, hypertension, type 2 diabetes, ischaemic heart disease, valvular heart disease, LVH on echocardiography, and renal impairment. Other categorical variables included place of care, follow-up care, NYHA class and peripheral oedema. Separate sub-group analyses for LVSD, diastolic HF on echocardiography and gender as well as their interactions with the presence or absence of AF were performed.

To understand the association between age, sex, the interaction between age and sex, rhythm status and risk of all-cause mortality at 1 year, we used the margins command in Stata to estimate margins of responses for specified values of covariates and present the results as a plot. Margins are statistics calculated from predictions of a previously fitted model (for example, a multivariable logistic regression) at fixed values of some covariates and averaging or otherwise integrating over the remaining covariates.

Sensitivity analyses were performed on models determined from the imputed data. Multiple imputations with chained equations were used to impute missing data. Five imputations were generated. Propensity score matching with nearest-neighbour matching was performed on the imputed cohort to create matched groups for presence or AF or sinus rhythm were used to estimate hazard ratios for mortality at 30-day, 1-year and full follow-up. We assessed the impact of heart rate as an effect modifier in a separate multivariable model. We employed the Stata module stddiff to estimate the standardised difference between groups It has been proposed that an absolute standardised difference of 0.10 or more indicates that covariates are imbalanced between groups [18].

Study findings are reported in accordance with the Strengthening the Reporting of Observational Studies in Epidemiology (STROBE) recommendations [19]. No ethics approval was needed for this analysis; the National Heart Failure Audit was conducted with the approval of the NHS Information Centre.

## Results

A total of 168,843 patients were admitted with AHF in England and Wales between January 2007 and December 2013. Supplemental Fig. 1 illustrates a flowchart of eligibility and exclusion of patients included in the analysis. After exclusions, 96,593 records were available for analyses, of which 44,642 patients were in SR (46.5%) and 51,951 (53.5%) in AF.

Table 1 outlines the distribution of patient’s characteristics by rhythm status. Patients with AF were significantly older than those with sinus rhythm [mean age 79.8 (79.7–80.0) vs 74.7 (74.5–74.7) years; *p* < 0.0001] and had a lower prevalence of diabetes, ischaemic heart disease and left ventricular systolic dysfunction (LVSD). The mean heart rate was greater in patients with AF 90 (89.5–90.3)] versus those with SR 84.0 (83.5–84.4); *p* < 0.0001.

**Table 1:** Distribution of patient characteristics by heart rhythm status

Variable		Atrial fibrillation	Sinus rhythm	
	Total^b^	51,951 (53.8)	44,642 (46.2)	
Age (years)	96,593	79.8 (79.7–80.0)	74.7 (74.5–74.7)	< 0.0001
Age categories (years)				
Min-54	5749/96,593	1521 (2.9)	4228 (9.5)	< 0.0001
55–64	8552/96,593	3299 (6.3)	5253 (11.8)	< 0.0001
65–74	18,524/96,593	8845 (17.0)	9679 (21.7)	< 0.0001
75–84	34,238/96,593	19,623 (37.8)	14,615 (32.7)	< 0.0001
85 +	29,530/96,593	18,663 (36.0)	10,867 (24.3)	< 0.0001
Sex				
Male	52,271/96,552	28,059 (54.0)	24,212 (54.3)	0.4925
Female	44,281/96,552	23,870 (46.0)	20,411 (45.7)	0.5277
Previous AMI	27,276/89,601	12,538 (26.3)	14,738 (35.1)	< 0.0001
History of diabetes	27,267/92,550	13,315 (26.9)	13,952 (32.4)	< 0.0001
History of hypertension	49,972/90,884	27,189 (55.9)	22,783 (53.9)	< 0.0001
History of IHD	42,387/90,874	21,690 (44.8)	20,697 (48.7)	< 0.0001
History of valvular heart disease	19,641/88,285	12,158 (25.8)	7483 (18.2)	< 0.0001
NYHA class				
Class I	5,294/88,888	2177 (4.6)	3117 (7.5)	< 0.0001
Class II	14,850/88,888	7348 (15.4)	7502 (18.2)	< 0.0001
Class III	39,210/88,888	21,316 (44.8)	17,894 (43.3)	< 0.0001
Class IV	29,534/88,888	16,717 (35.2)	12,817 (31.0)	< 0.0001
Peripheral oedema				
None	21,022/86,660	8965 (19.4)	12,057 (29.9)	< 0.0001
Mild	22,267/86,660	11,752 (25.4)	10,515 (26.0)	< 0.0001
Moderate	28,041/86,660	16,061 (34.7)	11,980 (29.7)	< 0.0001
Severe	15,330/86,660	9,513 (20.5)	5,817 (14.4)	< 0.0001
Moderate or severe LVSD^a^	54,475/79,724	27,577 (65.2)	26,898 (71.9)	< 0.0001
Heart rate (bpm)	20,976	90.0 (89.5–90.3)	84.0 (83.5–84.4)	< 0.0001

Column percentages are presented

*p* values from Student’s *t* tests, two-sample test of proportions or Chi-squared tests for differences in proportions for atrial fibrillation versus sinus rhythm respectively

*AMI* acute myocardial infarction, *IHD* ischaemic heart disease, *NYHA* New York Heart Association, *LVSD* left ventricle systolic dysfunction

^a^Only 20,976 participants had heart rate data recorded of which 9400 were in sinus rhythm and 11,576 were in AF

^b^Due to missing data, the percentages reported are from these totals

Table 2 illustrates differences in treatments, follow-up and mortality outcomes. Patients with AF were less likely to be prescribed an ACE inhibitor or ARB, but more likely to be prescribed a B-blocker or a diuretic. No statistically significant difference was observed in median length of stay between the two cohorts [9 (IQR 4–17) versus 8 (IQR 4–15) days in patients with AF and SR, respectively].

**Table 2:** Treatment, follow-up and mortality status by heart rhythm status

Variable		Atrial fibrillation	Sinus rhythm	*p* value
	Total			
Treatment medications				
MRA	29,402/80,939 (36.3)	15,776 (36.7)	13,626 (35.9)	0.017
ARB	11,651/74,027 (15.7)	6091 (15.5)	5560 (16.0)	0.058
ACE inhibitor	50,168/79,171 (63.4)	25,821 (61.6)	24,347 (65.4)	< 0.0001
ACE inhibitor OR ARB	61,165/80,903 (75.6)	31,598 (73.8)	29,567 (77.6)	< 0.0001
Beta-blocker	53,262/80,864 (65.9)	28,546 (66.3)	24,716 (65.3)	0.003
Thiazide diuretic	3647/79,869 (4.6)	2136 (5.0)	1511 (4.1)	< 0.0001
Digoxin	20,719/81,510 (25.4)	18,107 (40.9)	2612 (7.0)	< 0.0001
Loop diuretic	77,373/87,151 (88.8)	42,291 (90.9)	35,082 (86.4)	< 0.0001
Median length of stay in days (IQR)	8 (4–16)	9 (4–17)	8 (4–15)	< 0.0001
Discharge medications				
Beta-blocker	36,494/58,282 (62.6)	19,193 (62.7)	17,301 (62.6)	< 0.0001
Digoxin	15,134/58,514 (25.8)	13,185 (42.2)	1949 (7.2)	< 0.0001
Loop diuretic	55,140/62,531 (88.2)	29,820 (90.3)	25,320 (85.8)	< 0.0001
Thiazide	2558/58,385 (4.4)	1493 (4.8)	1065 (3.8)	< 0.0001
ARB	8305/54,425 (15.3)	4307 (15.0)	3998 (15.5)	< 0.0001
MRA	20,285/58,548 (34.6)	10,776 (35.1)	9509 (34.2)	0.025
ACE inhibitor	36,326/57,635 (63.0)	18,468 (61.2)	17,858 (65.1)	< 0.0001
ACE inhibitor OR ARB	44,125/58,709 (75.2)	22,541 (73.3)	21,584 (77.2)	< 0.0001
Main place of care				
Cardiology	43,290/95,963 (45.1)	21,952 (42.5)	21,338 (48.1)	< 0.0001
General medicine	42,203/95,963 (44.0)	23,777 (46.1)	18,426 (41.6)	< 0.0001
Other	10,470/95,963 (10.9)	5905 (11.4)	4565 (10.3)	0.0735
Follow-up				
Heart failure liaison service	43,807/88,852 (47.3)	22,303 (46.9)	21,504 (52.0)	< 0.0001
Palliative care	3,822/87,934 (4.4)	2242 (4.8)	1579 (3.8)	< 0.0001
Care of the elderly	12,279/88,951 (13.8)	6859 (14.4)	5420 (13.1)	< 0.0001
Cardiology	42,497/89,885 (49.3)	21,127 (44.0)	21,370 (51.0)	< 0.0001
GP follow-up	62,072/89,298 (69.5)	33,107 (69.2)	28,965 (69.8)	0.055

Column percentages are presented

*p* values from Student’s *t* tests, two-sample test of proportions or Chi-squared tests for differences in proportions for atrial fibrillation versus sinus rhythm respectively

*MRA* mineralocorticoid receptor antagonist, *ARB* angiotensin receptor blockers, *ACI* angiotensin-converting enzyme inhibitor, *IQR* interquartile range

Patients with SR were more likely to be managed in a cardiology ward and be followed up either by a heart failure service or a cardiologist (Table 2). In-hospital mortality was greater in patients with AF (11.5%) compared to those patients with sinus rhythm (8.6%; *p* < 0.0001) with similarly worse outcomes at 30 days (17.4% vs 13.5%, *p* < 0.0001) and 1 year (36.6% vs 30.0%, *p* < 0.0001). Figure 1 illustrates the crude Kaplan–Meier survival curves of both cohorts. Figure 2 in supplement shows variation in all-cause mortality by year of discharge from 2007 to 2013 and it remains highly consistent in AHF patients who have AF as compared to those who are in sinus rhythm.

**Fig. 2:**
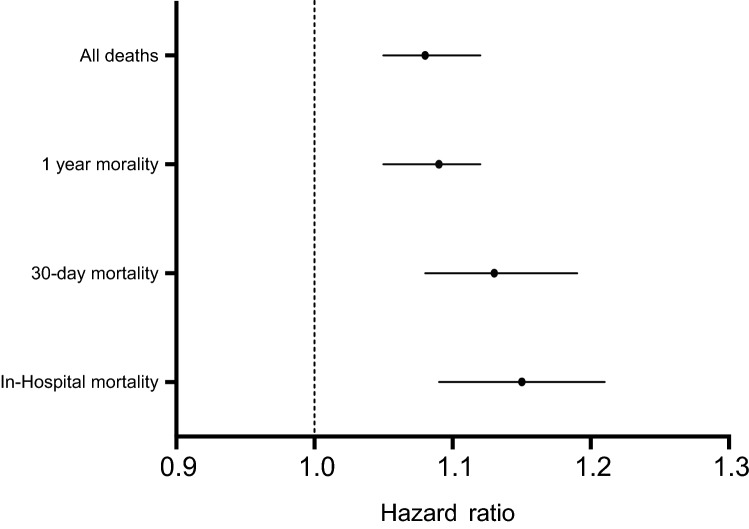
Hazard ratios for mortality from multivariate analysis for atrial fibrillation versus sinus rhythm at different time points (imputed data)

**Fig. 3:**
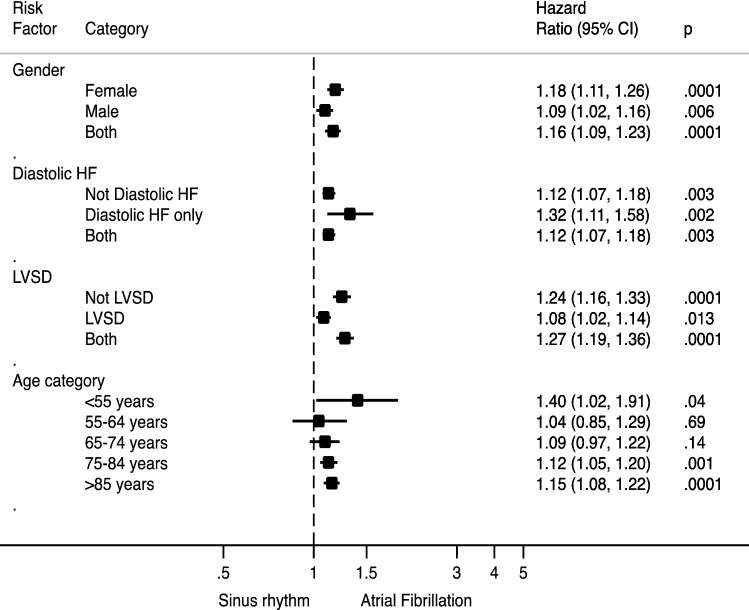
Sub-group analyses for risk of mortality at 30 days in AF versus sinus rhythm. *HF* heart failure, *LVSD* left ventricle systolic dysfunction

Multivariable analyses were undertaken to assess relationships between admission rhythm, clinical outcomes and in-hospital, 30-day (Supplement Table 1) and 1-year mortality (Tables 3, 4, 5; Fig. 2). The presence of AF was independently associated with mortality at all time points studied; in-hospital (HR 1.15, 95% CI 1.09–1.21, *p* < 0.0001), 30-day (HR 1.13, 95% CI 1.08–1.19, *p* < 0.0001) and 1-year (HR 1.09, 95% CI 1.05–1.12, *p* < 0.0001) outcomes. Finally, Fig. 3 illustrates the relationship between atrial fibrillation and mortality across gender, the presence of LVSD, diastolic dysfunction and age. AF was independently associated with worse 30-day mortality outcomes in all subgroups studied, except patients aged 55–64 and 65–74, where statistical trends that were not significant were observed (HR 1.04 95% CI 0.85–1.29, *p* = 0.69 and HR 1.09 95% CI 0.97–1.22).

**Table 3:** Multivariate analyses for relation between AF (versus sinus rhythm) and 1-year mortality on imputed data of patients recorded as HF, (*N* = 96,593)

	HR	95% CI		*p* value
Atrial fibrillation on ECG	1.09	1.05	1.12	< 0.001
Breathlessness				
NYHA I	1.00			
NYHA II	1.05	0.96	1.16	0.292
NYHA III	1.16	1.06	1.27	0.002
NYHA IV	1.30	1.19	1.42	0
Peripheral oedema				
None	1.00			
Mild	1.08	1.02	1.14	0.01
Moderate	1.23	1.17	1.30	< 0.0001
Severe	1.47	1.40	1.55	< 0.0001
Palliative care follow-up	2.54	2.35	2.74	< 0.0001
Heart failure liaison service	0.84	0.79	0.88	< 0.0001
GP follow-up	0.60	0.56	0.64	< 0.0001
Care of the elderly follow-up	0.80	0.76	0.85	< 0.0001
Cardiology follow-up	0.60	0.57	0.63	< 0.0001
Previous AMI	1.14	1.09	1.18	< 0.0001
History of diabetes	1.05	1.02	1.08	0.003
History of hypertension	0.93	0.90	0.95	< 0.0001
History of IHD	1.12	1.08	1.16	< 0.0001
History of valvular heart disease	1.24	1.19	1.29	< 0.0001
Age categories (years)				
Min-54	1.00			
55–64	1.43	1.27	1.59	< 0.0001
65–74	2.02	1.83	2.24	< 0.0001
75–84	2.74	2.47	3.04	< 0.0001
85 +	3.68	3.30	4.10	< 0.0001
Male	1.14	1.11	1.17	< 0.0001
LVH	0.86	0.81	0.92	< 0.0001
ACEi/ARB use	0.66	0.64	0.69	< 0.0001
Beta-blocker	0.78	0.75	0.82	< 0.0001
Thiazide	1.12	1.05	1.20	0.001
Loop diuretic	0.72	0.67	0.77	< 0.0001
Digoxin	0.89	0.85	0.92	< 0.0001
Renal failure	1.14	1.08	1.22	< 0.0001
Length of time in hospital (per 5 days)	1.02	1.02	1.03	< 0.0001

*HR* hazard ratio, *CI* confidence interval, *ECG* electrocardiograph, *NYHA* New York Heart Association, *AMI* acute myocardial infarction, *GP* general practitioner, *IHD* ischaemic heart disease, *LVH* left ventricular hypertrophy, *ARB* angiotensin receptor blockers, *ACI* angiotensin-converting enzyme inhibitor

**Table 4:** Multivariate analyses for relation between AF (versus sinus rhythm) and in-hospital mortality on imputed data of patients recorded as HF (*N* = 96,593)

	HR	95% CI		*p* value
Atrial fibrillation on ECG	1.15	1.09	1.21	< 0.0001
Breathlessness				
NYHA I	1.00			
NYHA II	1.01	0.87	1.18	0.896
NYHA III	1.10	0.96	1.27	0.18
NYHA IV	1.32	1.14	1.52	< 0.0001
Peripheral oedema				
None	1.00			
Mild	1.03	0.94	1.13	0.498
Moderate	1.09	1.01	1.18	0.023
Severe	1.33	1.22	1.45	< 0.0001
Palliative care follow-up	1.64	1.43	1.89	< 0.0001
Heart failure liaison service	0.51	0.44	0.59	< 0.0001
GP follow-up	0.23	0.19	0.27	< 0.0001
Care of the elderly follow-up	0.44	0.37	0.52	< 0.0001
Cardiology follow-up	0.33	0.28	0.39	< 0.0001
Previous AMI	1.05	0.99	1.11	0.123
History of diabetes	1.01	0.96	1.06	0.799
History of hypertension	0.97	0.92	1.01	0.156
History of IHD	1.08	1.03	1.13	0.003
History of valvular heart disease	1.16	1.10	1.23	< 0.0001
Age categories (years)				
Min-54	1.00			
55–64	1.38	1.13	1.70	0.002
65–74	1.88	1.55	2.28	< 0.0001
75–84	2.33	1.90	2.84	< 0.0001
85 +	2.72	2.21	3.36	< 0.0001
Male	1.06	1.02	1.11	0.003
LVH	0.88	0.80	0.97	0.01
ACEi/ARB use	0.77	0.72	0.83	< 0.0001
Beta-blocker	0.80	0.75	0.86	< 0.0001
Thiazide	0.91	0.78	1.06	0.205
Loop diuretic	0.70	0.65	0.76	< 0.0001
Digoxin	0.70	0.63	0.79	< 0.0001
Renal failure	1.15	1.06	1.25	0.003
Length of time in hospital (per 5 days)	1.02	1.02	1.03	< 0.0001

*HR* hazard ratio, *CI* confidence interval, *ECG* electrocardiograph, *NYHA* New York Heart Association, *AMI* acute myocardial infarction, GP general practitioner, *IHD* ischaemic heart disease, *LVH* left ventricular hypertrophy, *ARB* angiotensin receptor blockers, *ACI* angiotensin-converting enzyme inhibitor

**Table 5:** Multivariate analyses for relation between AF (versus sinus rhythm) and all deaths on imputed data of patients recorded as HF, (*N* = 96,593)

	HR	95% CI		*p* value
Atrial fibrillation on ECG	1.08	1.05	1.12	< 0.0001
Breathlessness				
NYHA I	1.00			
NYHA II	1.07	0.98	1.16	0.123
NYHA III	1.17	1.07	1.27	< 0.0001
NYHA IV	1.30	1.19	1.41	< 0.0001
Peripheral oedema				
None	1.00			
Mild	1.08	1.03	1.14	0.002
Moderate	1.23	1.18	1.29	< 0.0001
Severe	1.46	1.39	1.53	< 0.0001
Palliative care follow-up	2.48	2.28	2.69	< 0.0001
Heart failure liaison service	0.87	0.83	0.91	< 0.0001
GP follow-up	0.65	0.61	0.69	< 0.0001
Care of the elderly follow-up	0.83	0.78	0.87	< 0.0001
Cardiology follow-up	0.63	0.60	0.66	< 0.0001
Previous AMI	1.13	1.09	1.17	< 0.0001
History of diabetes	1.07	1.04	1.10	< 0.0001
History of hypertension	0.92	0.90	0.95	< 0.0001
History of IHD	1.13	1.09	1.17	< 0.0001
History of valvular heart disease	1.22	1.18	1.26	< 0.0001
Age categories (years)				
Min-54	1.00			
55–64	1.47	1.33	1.62	< 0.0001
65–74	2.11	1.93	2.31	< 0.0001
75–84	2.89	2.63	3.18	< 0.0001
85 +	4.00	3.63	4.41	< 0.0001
Male	1.14	1.11	1.17	< 0.0001
LVH	0.89	0.83	0.94	< 0.0001
ACEi/ARB use	0.68	0.65	0.70	< 0.0001
Beta-blocker	0.78	0.75	0.81	< 0.0001
Thiazide	1.13	1.06	1.20	< 0.0001
Loop diuretic	0.76	0.71	0.81	< 0.0001
Digoxin	0.90	0.87	0.93	< 0.0001
Renal failure	1.13	1.06	1.20	0.002
Length of time in hospital (per 5 days)	1.02	1.02	1.03	< 0.0001

*HR* hazard ratio, *CI* confidence interval, *ECG* electrocardiograph, *NYHA* New York Heart Association, *AMI* acute myocardial infarction, *GP* general practitioner, *IHD* ischaemic heart disease, *LVH* left ventricular hypertrophy, *ARB* angiotensin receptor blockers, *ACI* angiotensin-converting enzyme inhibitor

The impact of heart rate as an effect modifier is assessed in a separate multivariable model. Only around a quarter of the study population had heart rate data recorded. We assessed the standardised difference between groups (of individuals who had heart rate recorded (*n* = 20,976) and those who did not have heart recorded, *n* = 75,617) for both continuous and categorical variables. A table of these standardised differences are presented in Supplement Table 2. The impact of increased HR was assessed in a series of models in the 20,976 patients who had the data. Increased heart rate (HR increase by 10 beats per min) is associated with high inpatient deaths but lost its significance for long-term mortality (Supplement Tables 3, 4). AF is independently associated with adverse prognosis whether HR data are recorded or not in separate multivariable Cox regression models on imputed data. (Supplement Tables 5, 6). However, in a multivariable model from propensity score-matched (PSM) data in those with HR data, the adverse significant effect of AF was lost, likely due to type 2 errors from the small numbers present in model (Supplement table 7).

We assessed the interaction of gender or age with AF and its impact on 1-year mortality in a separate multivariable logistic regression analysis and results are presented in the form of margin plots (Supplement Fig. 3). Essentially, adverse effects of age and male gender on HF patients who have AF are minimal up to 60 years of age compared to those in sinus rhythm but increases clearly after it.

## Discussion

In this large multicentre national heart failure registry, we demonstrate that the presence of AF in patients admitted with an AHF is independently associated with an increased risk of in-hospital, 30-day and 1-year mortality. This relationship is observed in both HFREF and HFPEF, across genders and across different age groups in multivariate analyses.

These are the first data assessing the prognosis of AF in AHF during index hospital admission, 30 days and 1 year after discharge with detailed analysis in clinically important subgroups. The NHFA is one of the largest HF registries in the world providing more pragmatic real-world data as compared to post hoc analysis of clinical trials which are conducted in controlled clinical settings. No prior studies have evaluated the effect of AF in AHF patients across both genders and different age groups in the short and longer terms. Our study demonstrates significant differences in all clinical outcomes between patients with sinus rhythm and AF. Patients with HF who were found to be in AF have fewer comorbidities on presentation, prolonged hospital stay and worse short- and long-term mortalities in multivariable analysis. The results of this study are in contrast to previous smaller studies where AF was not associated with adverse long-term outcomes. For instance, in the Danish Investigations of Arrhythmia and Mortality on Dofetilide (DIAMOND) study (3587 patients), the authors reported similar in-hospital mortality between patients presenting in AF and sinus rhythm [12]. An analysis of the national GWTG-HF registry data (99,810 patients from 255 sites of US) also demonstrated a clear association between AF and mortality in hospitalised heart failure patients but no post-discharge follow-up data were available [7]. Our study represents a more contemporary cohort of patients (years of data collection 2007–2013) as compared to previously published research work (EHFS1 2000–2001, GWTG-HF 2005–2010, ASCEND-HF 2007–2010) [7, 11, 20]. Furthermore, our study also provides important information about processes of care in the treatment of these high-risk patients for the first time, such as their main place of care during hospital and post-discharge follow-up. It is pertinent to note that majority of these high-risk group patients with AF are neither treated in a cardiology ward (44%) nor followed up in cardiology clinics after discharge (47%). This finding is important and may provide a mechanism that contributes to our observations that patients with AF have adverse outcomes particularly in the longer term as previous work has demonstrated improved survival if HF patients are treated in cardiology wards or are followed up in either heart failure or cardiology clinics [21].

The relationship between AF and HF was first described in the literature almost 100 years ago [22]. This association could be explained to some extent by the presence of common risk factors such as age, diabetes, hypertension, high BMI, valvular, ischaemic and structural heart disease [23]. These risk factors contribute to myocardial cellular and extracellular damage, neurohormonal and electrophysiological changes which predispose the heart to AF and heart failure [24, 25]. The increased resting and exaggerated exercise heart rate response in AF predisposes to shorter diastolic filling time which leads to a reduction in cardiac output [26]. Furthermore, loss of effective atrial contraction also contributes to reduce diastolic filling. AF is considered the most common cause of tachycardia-induced cardiomyopathy. In a similar way, HF can increase the risk for the development of AF by raising cardiac filling pressures, autonomic and neuroendocrine dysregulation and increased interstitial fibrosis [27]. Despite the well-established association between these two conditions, less is known about the effects of AF particularly in HFPEF and HFREF on acute and long-term prognosis in patients hospitalised with heart failure.

Data that examine the relationship between AF and clinical outcomes in HF patients have many limitations and report inconsistent results. For instance, a retrospective analysis of SOLVD (Studies Of Left Ventricle Dysfunction) trial suggested that baseline AF was an independent predictor for all-cause mortality and the combined endpoint of death and readmission due to HF in patients who had ejection fraction < 35% [28]. Similar results were reported in the VALIANT trial (Valsartan in acute myocardial infarction) where AF was also associated with long-term mortality in acute myocardial infarction patients complicated by HF [28]. In contrast, a retrospective analysis of the Carvedilol or Metoprolol European Trial (COMET) data demonstrated that baseline AF significantly increased the risk of death and HF hospitalization in patients who had ejection fraction < 35%, although this association lost its significance in multivariate analysis [29].

Data around the prognostic impact of AF in patients with AHF are more limited. A retrospective analysis of Acute Study of Clinical Effectiveness of Nesiritide in Decompensated Heart Failure (ASCEND-HF) trial showed that the patients admitted to hospital with AHF, current or background history of AF are independently associated with lesser dyspnoea improvement, high mortality and morbidity (adjusted odds ratio 1.19, CI 1.02–1.38, *p* = 0.029) as compared to those who are in sinus rhythm [20]. Furthermore, in GWTG-HF study, the presence of AF was an independent predictor of in -hospital mortality and more prolonged index hospital admission (> 4 days) compared to sinus rhythm. However, in contrast to our analysis, no post-discharge mortality data were available. Moreover, the present analysis is more robust as compared to all other research on this subject as this study demonstrated that adverse effects of AF remain significant across gender and different age groups.

The relationship between AF and clinical outcomes in patients with HF may relate to the severity of LV dysfunction. For instance, Middlekauf et al. reported that AF was a stronger predictor of adverse outcomes (total mortality and sudden death) in patients with mild to moderate heart failure as compared to those with severe heart failure [30]. Similarly, Linssen et al. found that AF was associated with higher NT-proBNP levels, mortality and morbidity in HFPEF but not in HFREF patients [31]. In contrast, in a systematic review and meta-analysis of over 54,000 patients, Kotecha et al. reported that all-cause mortality was significantly higher in patients with AF and HFREF compared to HFPEF [32]. Our analysis has suggested that the adverse effect of AF remain significant in both HFPEF and HFREF in a cohort of 96,000 patients during hospital admission and 30 days after discharge.

The relationship between AF and outcomes is further complicated by the chronicity of AF. For example, new-onset and not chronic AF was an independent predictor of all-cause mortality in the EHFS1 and COMET studies [33]. As outlined, previous work has suggested that incident AF has a greater prognostic impact than chronic AF and established therapies for HF may reduce the incident AF risk. Retrospective analysis of large RCTs suggests that ACEI and ARB (angiotensin-converting enzyme inhibitors and angiotensin receptor blocker) can reduce the risk of incident AF in HF patients [34, 35]. However, this preventive role of ACEI/ARB is less evident in HFPEF [36, 37]. Initiation of beta-blocker (BB) therapy in HFREF patients who were pre-treated with ACEI/ARB was associated with one-third reduction of new-onset AF, [38] although BB does not reduce either mortality or hospital admission in patients who have HFREF and AF [38, 39]. Eplerenone also reduced the risk of incident AF in HF patients with LVEF < 35% when added to ACEI/ARB and BB [40]. In our study, a similar proportion of patients received BB at discharge in both groups (63%) but the AF cohort received less ACEI/ARB (76% versus 81%).

## Limitations

As with any observational study, there are a number of limitations. Whilst we have attempted to adjust for differences in clinical characteristics and patient demographics in the current work, the National Heart Failure audit does not collect data on co-morbid burden and frailty that is known to influence clinical outcomes in patients with AHF. Missing data is a common problem in large dataset studies which varied in extent depending on the study variable. However, we tried to approximate these values using multiple imputations to impute the missing variables. Prior history of AF was not recorded in NHFA dataset. Therefore, we are unable to differentiate between new-onset and chronic AF, as some previous studies report that the adverse prognostic impact of AF is limited to new-onset AF [7, 11]. Furthermore, data regarding the aetiology of heart failure are not recorded in this registry, and so it is unknown whether the prognostic impact of AF differs between the ischaemic and non-ischemic aetiologies of HF. Heart Failure with Mid-range ejection fraction (HFmrEF) is a recently defined entity in the European Society of Cardiology (ESC) 2016 HF guidelines, which encompass those patients who have a LVEF of 40–49%, elevated levels of natriuretic peptides and either LV hypertrophy, left atrium enlargement or diastolic dysfunction [41]. We were unable to analyse our dataset for this category as our data were collected before this publication [42].

## Conclusion

The current report, of approximately 100,000 unselected emergency hospital admissions due to heart failure, is the first to compare survival outcomes in AHF patients in the presence or absence of AF during an index hospital admission, at 30 days and 1 year after discharge, across gender and in different age groups. This study reveals that AF is associated with high mortality in AHF during hospital admission and up to 1 year after discharge after taking into account many potential confounders. Patients with AF in AHF are a high-risk cohort, although it is unclear whether targeting AF may improve outcomes in this cohort of patients, or whether it represents a marker of disease severity.

## Electronic supplementary material

Below is the link to the electronic supplementary material.

Supplementary material 1 (DOCX 5313 KB)
