# Supplementary material for: Does rhythm matter in acute heart failure? An insight from the British Society for Heart Failure National Audit
Source: Clin Res Cardiol. 2019 Apr 8;108(11):1276–86. doi: 10.1007/s00392-019-01463-5 (PMC6805810; doi:10.1007/s00392-019-01463-5)
Supplement: Supplementary file 1 — Supplementary material 1 (DOCX 5313 KB) [file 392_2019_1463_MOESM1_ESM.docx]

**Supplement**





**Supplement figure 1: Flow chart of eligibility and exclusion of patients included in analyses**


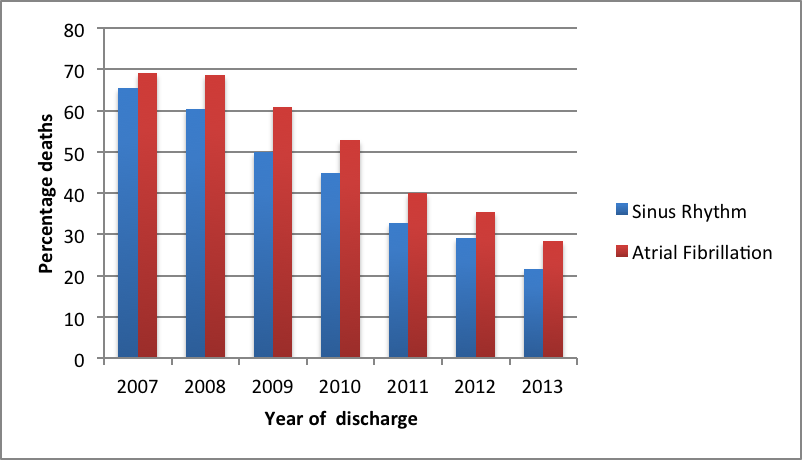


**Supplement Figure 2: Variation in all-cause mortality by year of discharge**


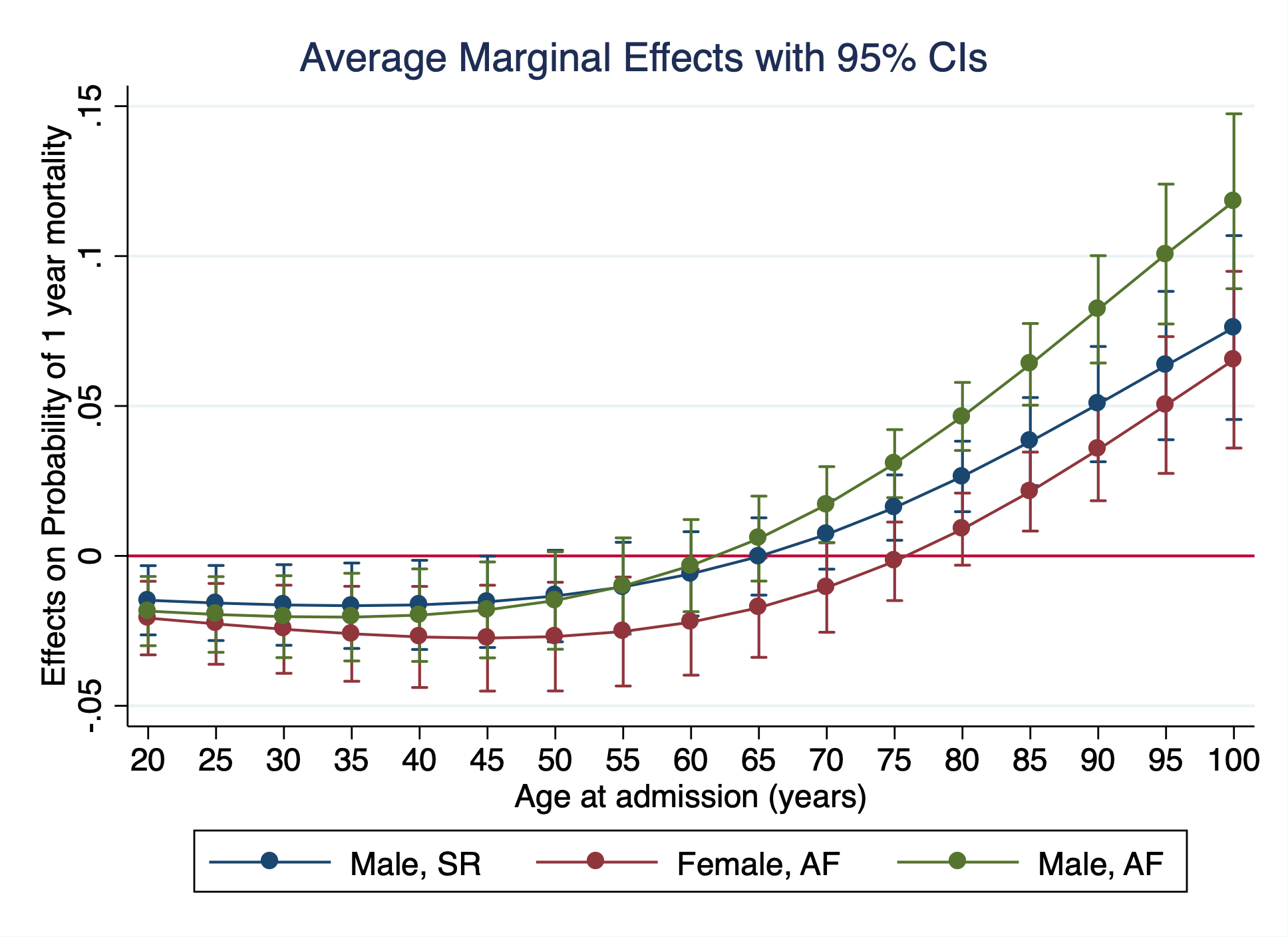


Supplement figure 3: Impact of gender and age on 1-year Mortality

Note: Women in Sinus rhythm are reference category

CI; Confidence interval, SR; Sinus Rhythms, AF; Atrial fibrillation,

Supplement table 1: Multivariate analyses for relation between AF (versus sinus rhythm) and 30-day mortality on imputed data of patients, (N = 96,593).

|  | HR | 95% CI | | Pvalue |
| --- | --- | --- | --- | --- |
| Atrial Fibrillation on ECG | 1.13 | 1.08 | 1.19 | <0.0001 |
| Breathlessness |  |  |  |  |
| NYHA I |  |  |  |  |
| NYHA II | 0.96 | 0.83 | 1.11 | 0.58 |
| NYHA III | 1.08 | 0.94 | 1.24 | 0.26 |
| NYHA IV | 1.31 | 1.15 | 1.48 | <0.0001 |
| Peripheral Oedema |  |  |  |  |
| None |  |  |  |  |
| Mild | 1.06 | 0.98 | 1.15 | 0.13 |
| Moderate | 1.20 | 1.12 | 1.28 | <0.0001 |
| Severe | 1.43 | 1.33 | 1.53 | <0.0001 |
| Palliative Care Follow-up | 2.53 | 2.30 | 2.79 | <0.0001 |
| Heart Failure Liaison service | 0.64 | 0.59 | 0.69 | <0.0001 |
| GP Follow-up | 0.38 | 0.34 | 0.42 | <0.0001 |
| Care of the Elderly Follow-up | 0.59 | 0.53 | 0.66 | <0.0001 |
| Cardiology Follow-up | 0.43 | 0.39 | 0.48 | <0.0001 |
| Previous AMI | 1.09 | 1.04 | 1.15 | 0.001 |
| History of Diabetes | 1.00 | 0.95 | 1.04 | 0.87 |
| History of Hypertension | 0.96 | 0.92 | 1.00 | 0.03 |
| History of IHD | 1.10 | 1.05 | 1.15 | <0.0001 |
| History of Valvular Heart disease | 1.20 | 1.15 | 1.26 | <0.0001 |
| Age Categories (years) |  |  |  |  |
| min-54 |  |  |  |  |
| 55-64 | 1.32 | 1.11 | 1.56 | 0.001 |
| 65-74 | 1.90 | 1.64 | 2.21 | <0.0001 |
| 75-84 | 2.37 | 2.03 | 2.76 | <0.0001 |
| 85+ | 2.90 | 2.48 | 3.40 | <0.0001 |
| Male | 1.11 | 1.07 | 1.15 | <0.0001 |
| LVH | 0.85 | 0.78 | 0.93 | <0.0001 |
| ACEi/ARB use | 0.66 | 0.62 | 0.70 | <0.0001 |
| Beta-blocker | 0.79 | 0.75 | 0.83 | <0.0001 |
| Thiazide | 0.99 | 0.90 | 1.10 | 0.91 |
| Loop diuretic | 0.65 | 0.61 | 0.70 | <0.0001 |
| Digoxin | 0.77 | 0.71 | 0.83 | <0.0001 |
| Renal failure | 1.17 | 1.05 | 1.29 | 0.01 |
| Length of time in hospital (per 5 days) | 1.02 | 1.02 | 1.03 | <0.0001 |

HR; Hazard ratio, CI; Confidence interval, ECG; Electro cardio graph, NYHA; New York Heart Association, AMI; Acute Myocardial Infarction, GP; General practitioner, IHD; Ischaemic Heart disease, LVH; Left Ventricular hypertrophy, ARB; Angiotensin receptor blockers, ACI; Angiotensin converting enzyme inhibitor.

Supplement Table 2: Standardised differences between those with Heart rate (HR) data (n=20,976) and those without HR data present (n=75,617)

| Atrial Fibrillation | | | | Sinus Rhythm | | |
| --- | --- | --- | --- | --- | --- | --- |
|  | Missing HR data | HR data present |  | Missing HR data | HR data present |  |
|  | 40,372 | 11,576 |  | 35,242 | 9400 |  |
|  | Mean or N SD or (%) | Mean or N SD or (%) | Std Diff | Mean or N SD or (%) | Mean or N SD or (%) | Std Diff |
| Age (years) | 79.59 10.715 | 80.57 10.29 | 0.09254 | 74.47 13.69 | 75.33 13.584 | 0.06274 |
| Gender |  |  |  |  |  |  |
| Female | 18490 (45.8) | 5380 (46.5) | 0.01376 | 15998 (45.4) | 4413 (47.0) | 0.03102 |
| Male | 21869 (54.2) | 6190 (53.5) |  | 19228 (54.6) | 4984 (53.0) |  |
| Previous AMI |  |  |  |  |  |  |
| No | 26713 (73.1) | 8405 (75.8) | 0.0621 | 21012 (63.9) | 6195 (68.2) | 0.09056 |
| Yes | 9850 (26.9) | 2688 (24.2) |  | 11852 (36.1) | 2886 (31.8) |  |
| Previous DM |  |  |  |  |  |  |
| No | 28006 (73.5) | 8122 (71.7) | 0.03934 | 22963 (67.8) | 6192 (67.1) | 0.01331 |
| Yes | 10112 (26.5) | 3203 (28.3) |  | 10922 (32.2) | 3030 (32.9) |  |
| Previous Hypertension |  |  |  |  |  |  |
| No | 16734 (44.8) | 4694 (41.7) | 0.06196 | 15421 (46.6) | 4063 (44.3) | 0.04491 |
| Yes | 20631 (55.2) | 6558 (58.3) |  | 17684 (53.4) | 5099 (55.7) |  |
| Previous IHD |  |  |  |  |  |  |
| No | 20309 (54.5) | 6390 (57.4) | 0.0572 | 16890 (50.6) | 4898 (53.7) | 0.06281 |
| Yes | 16940 (45.5) | 4750 (42.6) |  | 16482 (49.4) | 4215 (46.3) |  |
| Age category |  |  |  |  |  |  |
| 45- | 1240 ( 3.1) | 281 ( 2.4) | 0.12733 | 3437 ( 9.8) | 791 ( 8.4) | 0.10211 |
| 55- | 2717 ( 6.7) | 582 ( 5.0) |  | 4232 (12.0) | 1021 (10.9) |  |
| 65- | 7048 (17.5) | 1797 (15.5) |  | 7729 (21.9) | 1950 (20.7) |  |
| 75- | 15333 (38.0) | 4290 (37.1) |  | 11569 (32.8) | 3046 (32.4) |  |
| 85- | 14037 (34.8) | 4626 (40.0) |  | 8275 (23.5) | 2592 (27.6) |  |
| MRA |  |  |  |  |  |  |
| No | 21837 (64.6) | 5368 (58.5) | 0.12557 | 19781 (65.4) | 4551 (59.1) | 0.13083 |
| Yes | 11968 (35.4) | 3808 (41.5) |  | 10471 (34.6) | 3155 (40.9) |  |
| ARB |  |  |  |  |  |  |
| No | 26421 (84.7) | 6783 (83.8) | 0.0255 | 23472 (84.3) | 5700 (82.8) | 0.03927 |
| Yes | 4776 (15.3) | 1315 (16.2) |  | 4378 (15.7) | 1182 (17.2) |  |
| ACE inhibitor |  |  |  |  |  |  |
| No | 12789 (38.6) | 3318 (37.6) | 0.02078 | 10293 (34.6) | 2603 (34.7) | 0.0032 |
| Yes | 20319 (61.4) | 5502 (62.4) |  | 19459 (65.4) | 4888 (65.3) |  |
| Beta blocker |  |  |  |  |  |  |
| No | 12154 (36.0) | 2335 (25.1) | 0.23915 | 10916 (36.3) | 2197 (28.3) | 0.1714 |

HR; Hazard ratio, CI; Confidence interval, ECG; Electro cardio graph, NYHA; New York Heart Association, AMI; Acute Myocardial Infarction, GP; General practitioner, IHD; Ischaemic Heart disease, LVH; Left Ventricular hypertrophy, ARB; Angiotensin receptor blockers, ACI; Angiotensin converting enzyme inhibitor.

Supplement table 3: Multivariable Cox regression analyses for in-hospital mortality in patients with heart rate (HR) data recorded, (n= 20,956)

| **Model 1** | | | | | **Model 2: (Model 1 + Heart Rate)** | | | |
| --- | --- | --- | --- | --- | --- | --- | --- | --- |
|  | Hazard Ratio | 95% Confidence interval | | P value | Hazard Ratio | 95% Confidence Interval | | P value |
| Atrial Fibrillation on ECG | 1.14 | 1.05 | 1.24 | 0.002 | 1.12 | 1.03 | 1.22 | 0.007 |
| Heart rate (per 10 bpm) |  |  |  |  | 1.04 | 1.02 | 1.06 | <0.0001 |
| Breathlessness |  |  |  |  |  |  |  |  |
| NYHA I | 1.00 |  |  |  | 1.00 |  |  |  |
| NYHA II | 1.11 | 0.86 | 1.43 | 0.425 | 1.12 | 0.87 | 1.45 | 0.382 |
| NYHA III | 1.09 | 0.87 | 1.37 | 0.456 | 1.10 | 0.87 | 1.38 | 0.437 |
| NYHA IV | 1.26 | 1.01 | 1.57 | 0.037 | 1.26 | 1.01 | 1.56 | 0.041 |
| Peripheral Oedema |  |  |  |  |  |  |  |  |
| None | 1.00 |  |  |  | 1.00 |  |  |  |
| Mild | 1.01 | 0.89 | 1.15 | 0.861 | 1.02 | 0.89 | 1.16 | 0.826 |
| Moderate | 1.07 | 0.93 | 1.24 | 0.317 | 1.08 | 0.94 | 1.25 | 0.267 |
| Severe | 1.26 | 1.09 | 1.47 | 0.002 | 1.29 | 1.10 | 1.50 | 0.001 |
| Palliative Care Follow-up | 1.58 | 1.31 | 1.91 | <0.0001 | 1.57 | 1.30 | 1.89 | <0.0001 |
| Heart Failure Liaison service | 0.56 | 0.46 | 0.67 | <0.0001 | 0.56 | 0.46 | 0.68 | <0.0001 |
| GP Follow-up | 0.23 | 0.18 | 0.29 | <0.0001 | 0.23 | 0.18 | 0.29 | <0.0001 |
| Care of the Elderly Follow-up | 0.55 | 0.43 | 0.71 | <0.0001 | 0.55 | 0.43 | 0.71 | <0.0001 |
| Cardiology Follow-up | 0.46 | 0.35 | 0.60 | <0.0001 | 0.46 | 0.35 | 0.60 | <0.0001 |
| Previous AMI | 1.03 | 0.93 | 1.13 | 0.601 | 1.04 | 0.94 | 1.14 | 0.466 |
| History of Diabetes | 1.03 | 0.94 | 1.13 | 0.492 | 1.04 | 0.95 | 1.14 | 0.381 |
| History of Hypertension | 0.91 | 0.83 | 1.00 | 0.039 | 0.91 | 0.84 | 1.00 | 0.051 |
| History of IHD | 1.11 | 1.01 | 1.22 | 0.031 | 1.12 | 1.02 | 1.22 | 0.022 |
| History of Valvular Heart disease | 1.12 | 1.02 | 1.24 | 0.021 | 1.14 | 1.03 | 1.26 | 0.011 |
| Age Categories (years) |  |  |  |  |  |  |  |  |
| min-54 | 1.00 |  |  |  | 1.00 |  |  |  |
| 55-64 | 1.75 | 0.93 | 3.31 | 0.083 | 1.76 | 0.93 | 3.33 | 0.081 |
| 65-74 | 2.81 | 1.56 | 5.06 | 0.001 | 2.88 | 1.60 | 5.20 | <0.0001 |
| 75-84 | 3.27 | 1.84 | 5.82 | <0.0001 | 3.37 | 1.90 | 5.99 | <0.0001 |
| 85+ | 3.73 | 2.06 | 6.75 | <0.0001 | 3.87 | 2.14 | 6.99 | <0.0001 |
| Male | 0.98 | 0.90 | 1.07 | 0.666 | 1.00 | 0.91 | 1.09 | 0.967 |
| LVH | 0.93 | 0.78 | 1.12 | 0.459 | 0.94 | 0.78 | 1.12 | 0.495 |
| ACEi/ARB use | 0.84 | 0.75 | 0.94 | 0.003 | 0.84 | 0.75 | 0.94 | 0.003 |
| Beta-blocker | 0.67 | 0.59 | 0.77 | <0.0001 | 0.67 | 0.58 | 0.77 | <0.0001 |
| Thiazide | 0.94 | 0.70 | 1.26 | 0.643 | 0.95 | 0.70 | 1.27 | 0.698 |
| Loop diuretic | 0.71 | 0.63 | 0.81 | <0.0001 | 0.72 | 0.63 | 0.81 | <0.0001 |
| Digoxin | 0.86 | 0.74 | 1.01 | 0.067 | 0.85 | 0.72 | 0.99 | 0.043 |
| Renal Failure | 1.72 | 1.51 | 1.97 | <0.0001 | 1.75 | 1.54 | 1.99 | <0.0001 |
| Length of time in hospital (per 5 days) | 1.03 | 1.02 | 1.05 | <0.0001 | 1.04 | 1.02 | 1.05 | <0.0001 |

HR; Hazard ratio, CI; Confidence interval, ECG; Electro cardio graph, NYHA; New York Heart Association, AMI; Acute Myocardial Infarction, GP; General practitioner, IHD; Ischaemic Heart disease, LVH; Left Ventricular hypertrophy, ARB; Angiotensin receptor blockers, ACI; Angiotensin converting enzyme inhibitor. Supplement table 4: Multivariable Cox regression analyses for all deaths in patients with Heart Rate (HR) data recorded, (n= 20,956)

| Model 1 | | | | | Model 2: (Model 1 + Heart rate) | | | |
| --- | --- | --- | --- | --- | --- | --- | --- | --- |
|  | Hazard ratio | 95% Confidence Interval | | P value | Hazard ratio | 95% Confidence Interval | | P value |
| Atrial Fibrillation on ECG | 1.08 | 1.02 | 1.15 | 0.009 | 1.08 | 1.01 | 1.14 | 0.016 |
| Heart rate (per 10 bpm) |  |  |  |  | 1.01 | 0.99 | 1.02 | 0.286 |
| Breathlessness |  |  |  |  |  |  |  |  |
| NYHA I |  |  |  |  | 1.00 |  |  |  |
| NYHA II | 1.10 | 0.95 | 1.27 | 0.182 | 1.08 | 0.92 | 1.26 | 0.337 |
| NYHA III | 1.15 | 1.00 | 1.31 | 0.047 | 1.12 | 0.97 | 1.29 | 0.13 |
| NYHA IV | 1.18 | 1.03 | 1.35 | 0.015 | 1.16 | 1.02 | 1.33 | 0.029 |
| Peripheral Oedema |  |  |  |  |  |  |  |  |
| None | 1.00 |  |  |  | 1.00 |  |  |  |
| Mild | 1.02 | 0.93 | 1.12 | 0.724 | 1.02 | 0.92 | 1.13 | 0.656 |
| Moderate | 1.13 | 1.05 | 1.22 | 0.002 | 1.13 | 1.03 | 1.23 | 0.008 |
| Severe | 1.37 | 1.25 | 1.50 | <0.0001 | 1.37 | 1.25 | 1.51 | <0.0001 |
| Palliative Care Follow-up | 2.66 | 2.42 | 2.92 | <0.0001 | 2.62 | 2.35 | 2.91 | <0.0001 |
| Heart Failure Liaison service | 0.83 | 0.77 | 0.89 | <0.0001 | 0.82 | 0.77 | 0.89 | <0.0001 |
| GP Follow-up | 0.53 | 0.48 | 0.59 | <0.0001 | 0.54 | 0.49 | 0.60 | <0.0001 |
| Care of the Elderly Follow-up | 0.82 | 0.75 | 0.89 | <0.0001 | 0.81 | 0.74 | 0.89 | <0.0001 |
| Cardiology Follow-up | 0.63 | 0.58 | 0.69 | <0.0001 | 0.63 | 0.57 | 0.69 | <0.0001 |
| Previous AMI | 1.07 | 1.00 | 1.14 | 0.06 | 1.07 | 1.00 | 1.15 | 0.05 |
| History of Diabetes | 1.02 | 0.96 | 1.08 | 0.549 | 1.02 | 0.96 | 1.09 | 0.539 |
| History of Hypertension | 0.91 | 0.86 | 0.96 | 0.001 | 0.91 | 0.86 | 0.97 | 0.003 |
| History of IHD | 1.14 | 1.08 | 1.21 | <0.0001 | 1.15 | 1.08 | 1.22 | <0.0001 |
| History of Valvular Heart disease | 1.22 | 1.14 | 1.31 | <0.0001 | 1.24 | 1.16 | 1.32 | <0.0001 |
| Age Categories (years) |  |  |  |  |  |  |  |  |
| min-54 | 1.00 |  |  |  | 1.00 |  |  |  |
| 55-64 | 1.90 | 1.43 | 2.52 | <0.0001 | 1.89 | 1.42 | 2.51 | <0.0001 |
| 65-74 | 2.58 | 1.96 | 3.39 | <0.0001 | 2.56 | 1.95 | 3.36 | <0.0001 |
| 75-84 | 3.60 | 2.76 | 4.70 | <0.0001 | 3.60 | 2.76 | 4.69 | <0.0001 |
| 85+ | 4.78 | 3.68 | 6.21 | <0.0001 | 4.76 | 3.67 | 6.19 | <0.0001 |
| Male | 1.07 | 1.01 | 1.14 | 0.014 | 1.09 | 1.02 | 1.15 | 0.005 |
| LVH | 0.94 | 0.85 | 1.05 | 0.277 | 0.94 | 0.85 | 1.04 | 0.256 |
| ACEi/ARB use | 0.75 | 0.71 | 0.80 | <0.0001 | 0.75 | 0.70 | 0.80 | <0.0001 |
| Beta-blocker | 0.73 | 0.69 | 0.78 | <0.0001 | 0.71 | 0.66 | 0.77 | <0.0001 |
| Thiazide | 1.12 | 1.00 | 1.26 | 0.047 | 1.09 | 0.95 | 1.25 | 0.197 |
| Loop diuretic | 0.68 | 0.62 | 0.76 | 0 | 0.70 | 0.62 | 0.79 | <0.0001 |
| Digoxin | 0.91 | 0.84 | 0.99 | 0.019 | 0.93 | 0.85 | 1.01 | 0.088 |
| Renal Failure | 1.73 | 1.59 | 1.87 | <0.0001 | 1.71 | 1.57 | 1.86 | <0.0001 |
| Length of time in hospital (per 5 days) | 1.05 | 1.04 | 1.06 | <0.0001 | 1.04 | 1.03 | 1.06 | <0.0001 |

HR; Hazard ratio, CI; Confidence interval, ECG; Electro cardio graph, NYHA; New York Heart Association, AMI; Acute Myocardial Infarction, GP; General practitioner, IHD; Ischaemic Heart disease, LVH; Left Ventricular hypertrophy, ARB; Angiotensin receptor blockers, ACI; Angiotensin converting enzyme inhibitor.

Supplement table 5: Multivariable Cox regression analyses for the association between presence of AF vs. sinus rhythm and 1-year mortality in patients with AHF who did not have Heart rate (HR) data recorded, (n=75,617)

|  | Hazard Ratio | 95% Confidence Interval | | P value |
| --- | --- | --- | --- | --- |
| Atrial Fibrillation on ECG | 1.09 | 1.05 | 1.12 | <0.0001 |
| Breathlessness |  |  |  |  |
| NYHA I | 1.00 |  |  |  |
| NYHA II | 1.05 | 0.96 | 1.16 | 0.292 |
| NYHA III | 1.16 | 1.06 | 1.27 | 0.002 |
| NYHA IV | 1.30 | 1.19 | 1.42 | <0.0001 |
| Peripheral Oedema |  |  |  |  |
| None | 1.00 |  |  |  |
| Mild | 1.08 | 1.02 | 1.14 | 0.01 |
| Moderate | 1.23 | 1.17 | 1.30 | <0.0001 |
| Severe | 1.47 | 1.40 | 1.55 | <0.0001 |
| Palliative Care Follow-up | 2.54 | 2.35 | 2.74 | <0.0001 |
| Heart Failure Liaison service | 0.84 | 0.79 | 0.88 | <0.0001 |
| GP Follow-up | 0.60 | 0.56 | 0.64 | <0.0001 |
| Care of the Elderly Follow-up | 0.80 | 0.76 | 0.85 | <0.0001 |
| Cardiology Follow-up | 0.60 | 0.57 | 0.63 | <0.0001 |
| Previous AMI | 1.14 | 1.09 | 1.18 | <0.0001 |
| History of Diabetes | 1.05 | 1.02 | 1.08 | 0.003 |
| History of Hypertension | 0.93 | 0.90 | 0.95 | <0.0001 |
| History of IHD | 1.12 | 1.08 | 1.16 | <0.0001 |
| History of Valvular Heart disease | 1.24 | 1.19 | 1.29 | <0.0001 |
| Age Categories (years) |  |  |  |  |
| min-54 | 1.00 |  |  |  |
| 55-64 | 1.43 | 1.27 | 1.59 | <0.0001 |
| 65-74 | 2.02 | 1.83 | 2.24 | <0.0001 |
| 75-84 | 2.74 | 2.47 | 3.04 | <0.0001 |
| 85+ | 3.68 | 3.30 | 4.10 | <0.0001 |
| Male | 1.14 | 1.11 | 1.17 | <0.0001 |
| LVH | 0.86 | 0.81 | 0.92 | <0.0001 |
| ACEi/ARB use | 0.66 | 0.64 | 0.69 | <0.0001 |
| Beta-blocker | 0.78 | 0.75 | 0.82 | <0.0001 |
| Thiazide | 1.12 | 1.05 | 1.20 | 0.001 |
| Loop diuretic | 0.72 | 0.67 | 0.77 | <0.0001 |
| Digoxin | 0.89 | 0.85 | 0.92 | <0.0001 |
| Renal Failure | 1.14 | 1.08 | 1.22 | <0.0001 |
| Length of time in hospital (per 5 days) | 1.02 | 1.02 | 1.03 | <0.0001 |

HR; Hazard ratio, CI; Confidence interval, ECG; Electro cardio graph, NYHA; New York Heart Association, AMI; Acute Myocardial Infarction, GP; General practitioner, IHD; Ischaemic Heart disease, LVH; Left Ventricular hypertrophy, ARB; Angiotensin receptor blockers, ACI; Angiotensin converting enzyme inhibitor.

Supplement table 6: Multivariable Cox regression analyses for the association between presence of AF vs. sinus rhythm and 1-year mortality in patients with heart rate (HR) data recorded (n= 20,956)

|  | Hazard Ratio | 95% Confidence Interval | | P value |
| --- | --- | --- | --- | --- |
| Atrial Fibrillation on ECG | 1.08 | 1.02 | 1.14 | 0.014 |
| Breathlessness |  |  |  |  |
| NYHA I |  |  |  |  |
| NYHA II | 1.07 | 0.92 | 1.25 | 0.374 |
| NYHA III | 1.11 | 0.96 | 1.29 | 0.141 |
| NYHA IV | 1.16 | 1.02 | 1.33 | 0.029 |
| Peripheral Oedema |  |  |  |  |
| None | 1.00 |  |  |  |
| Mild | 1.03 | 0.93 | 1.14 | 0.627 |
| Moderate | 1.13 | 1.03 | 1.23 | 0.008 |
| Severe | 1.38 | 1.25 | 1.52 | <0.0001 |
| Palliative Care Follow-up | 2.62 | 2.36 | 2.92 | <0.0001 |
| Heart Failure Liaison service | 0.83 | 0.77 | 0.89 | <0.0001 |
| GP Follow-up | 0.54 | 0.49 | 0.60 | <0.0001 |
| Care of the Elderly Follow-up | 0.81 | 0.73 | 0.88 | <0.0001 |
| Cardiology Follow-up | 0.63 | 0.57 | 0.69 | <0.0001 |
| Previous AMI | 1.07 | 1.00 | 1.15 | 0.062 |
| History of Diabetes | 1.02 | 0.95 | 1.08 | 0.603 |
| History of Hypertension | 0.91 | 0.86 | 0.97 | 0.002 |
| History of IHD | 1.15 | 1.08 | 1.22 | <0.0001 |
| History of Valvular Heart disease | 1.23 | 1.15 | 1.32 | <0.0001 |
| Age Categories (years) |  |  |  |  |
| min-54 | 1.00 |  |  |  |
| 55-64 | 1.88 | 1.42 | 2.50 | <0.0001 |
| 65-74 | 2.57 | 1.95 | 3.38 | <0.0001 |
| 75-84 | 3.60 | 2.75 | 4.71 | <0.0001 |
| 85+ | 4.75 | 3.64 | 6.19 | <0.0001 |
| Male | 1.08 | 1.02 | 1.14 | 0.012 |
| LVH | 0.94 | 0.84 | 1.04 | 0.221 |
| ACEi/ARB use | 0.75 | 0.69 | 0.80 | <0.0001 |
| Beta-blocker | 0.71 | 0.66 | 0.77 | <0.0001 |
| Thiazide | 1.09 | 0.95 | 1.25 | 0.215 |
| Loop diuretic | 0.70 | 0.62 | 0.79 | <0.0001 |
| Digoxin | 0.93 | 0.85 | 1.02 | 0.104 |
| Renal Failure | 1.71 | 1.57 | 1.86 | <0.0001 |
| Length of time in hospital (per 5 days) | 1.04 | 1.03 | 1.06 | <0.0001 |

Supplement table 7: Multivariable Cox regression analyses for the association between presence of AF (n=5,866) vs those with sinus rhythm (n=5,866) and 1-year mortality in patients with heart rate (HR) after Propensity Score Matching (PSM) ^*^.

|  | Hazard Ratio | 95% Confidence Interval | | P value |
| --- | --- | --- | --- | --- |
| Atrial Fibrillation on ECG | 1.05 | 0.91 | 1.21 | 0.516 |
| Heart rate (per 10 bpm) | 1.00 | 1.00 | 1.00 | 0.805 |
| Peripheral Oedema |  |  |  |  |
| None | 1.00 |  |  |  |
| Mild | 1.05 | 0.85 | 1.29 | 0.679 |
| Moderate | 1.00 | 0.81 | 1.23 | 0.989 |
| Severe | 1.45 | 1.20 | 1.75 | <0.0001 |
| Palliative Care Follow-up | 3.12 | 2.20 | 4.41 | <0.0001 |
| Heart Failure Liaison service | 1.00 | 0.85 | 1.18 | 0.985 |
| GP Follow-up | 0.51 | 0.42 | 0.62 | <0.0001 |
| Care of the Elderly Follow-up | 0.88 | 0.73 | 1.07 | 0.195 |
| Cardiology Follow-up | 0.70 | 0.60 | 0.82 | <0.0001 |
| Previous AMI | 1.19 | 0.98 | 1.45 | 0.076 |
| History of Diabetes | 0.83 | 0.70 | 0.97 | 0.024 |
| History of Hypertension | 0.90 | 0.77 | 1.06 | 0.217 |
| History of IHD | 1.08 | 0.89 | 1.30 | 0.437 |
| History of Valvular Heart disease | 1.22 | 1.05 | 1.43 | 0.011 |
| Age Categories (years) |  |  |  |  |
| min-54 |  |  |  |  |
| 55-64 | 1.24 | 0.66 | 2.35 | 0.507 |
| 65-74 | 1.93 | 1.06 | 3.49 | 0.03 |
| 75-84 | 2.70 | 1.52 | 4.81 | 0.001 |
| 85+ | 3.95 | 2.18 | 7.18 | <0.0001 |
| Male | 1.16 | 1.00 | 1.34 | 0.052 |
| ACEi/ARB use | 0.71 | 0.61 | 0.83 | <0.0001 |
| Beta-blocker | 0.67 | 0.57 | 0.80 | <0.0001 |
| Thiazide | 1.08 | 0.81 | 1.43 | 0.622 |
| Loop diuretic | 0.67 | 0.47 | 0.96 | 0.03 |
| Digoxin | 0.90 | 0.74 | 1.10 | 0.307 |
| Renal Failure | 1.98 | 1.57 | 2.51 | <0.0001 |
| Length of time in hospital (per 5 days) | 1.04 | 1.01 | 1.06 | 0.003 |

HR; Hazard ratio, CI; Confidence interval, ECG; Electro cardio graph, NYHA; New York Heart Association, AMI; Acute Myocardial Infarction, GP; General practitioner, IHD; Ischaemic Heart disease, LVH; Left Ventricular hypertrophy, ARB; Angiotensin receptor blockers, ACI; Angiotensin converting enzyme inhibitor. ^*^PSM matching (by presence of sinus rhythm or AF) conducted in those individuals with heart rate data available (n= 20,956). The final sample size after 1:1 matching was N= 11,732.
